# The Datafication of Hate: Expectations and Challenges in Automated Hate Speech Monitoring

**DOI:** 10.3389/fdata.2020.00003

**Published:** 2020-02-05

**Authors:** Salla-Maaria Laaksonen, Jesse Haapoja, Teemu Kinnunen, Matti Nelimarkka, Reeta Pöyhtäri

**Affiliations:** ^1^Centre for Consumer Society Research, University of Helsinki, Helsinki, Finland; ^2^Department of Computer Science, Aalto University, Espoo, Finland; ^3^Social Psychology, Faculty of Social Sciences, University of Helsinki, Helsinki, Finland; ^4^Futurice, Helsinki, Finland; ^5^Centre for Research Methods and Social Statistics, University of Helsinki, Helsinki, Finland; ^6^Department of Language and Communication Studies, University of Jyväskylä, Jyväskylä, Finland; ^7^Research Centre for Journalism, Media and Communication, Faculty of Information Technology and Communication Sciences, Tampere University, Tampere, Finland

**Keywords:** hate speech, machine learning, algorithmic system, data science, social media, politics

## Abstract

Hate speech has been identified as a pressing problem in society and several automated approaches have been designed to detect and prevent it. This paper reports and reflects upon an action research setting consisting of multi-organizational collaboration conducted during Finnish municipal elections in 2017, wherein a technical infrastructure was designed to automatically monitor candidates' social media updates for hate speech. The setting allowed us to engage in a 2-fold investigation. First, the collaboration offered a unique view for exploring how hate speech emerges as a technical problem. The project developed an adequately well-working algorithmic solution using supervised machine learning. We tested the performance of various feature extraction and machine learning methods and ended up using a combination of Bag-of-Words feature extraction with Support-Vector Machines. However, an automated approach required heavy simplification, such as using rudimentary scales for classifying hate speech and a reliance on word-based approaches, while in reality hate speech is a linguistic and social phenomenon with various tones and forms. Second, the action-research-oriented setting allowed us to observe affective responses, such as the hopes, dreams, and fears related to machine learning technology. Based on participatory observations, project artifacts and documents, interviews with project participants, and online reactions to the detection project, we identified participants' aspirations for effective automation as well as the level of neutrality and objectivity introduced by an algorithmic system. However, the participants expressed more critical views toward the system after the monitoring process. Our findings highlight how the powerful expectations related to technology can easily end up dominating a project dealing with a contested, topical social issue. We conclude by discussing the problematic aspects of datafying hate and suggesting some practical implications for hate speech recognition.

## Introduction

Discriminating, hateful speech online, often targeting specific groups and minorities, has become a pressing problem in societies (e.g., Sharma, 2013; Gagliardone et al., 2015; Hardaker and McGlashan, 2016; Baider et al., 2017; Matamoros-Fernández, 2017). Hateful speech potentially creates enmities, silences debates, and marginalizes individuals and groups from participation online. What is challenging is that “hate speech” now refers to a variety of speech acts and other ill-behaviors taking place online, ranging from penal criminal acts to speech that is uncivil and disturbing, and yet tolerated (e.g., Baider et al., 2017). This definitional difficulty is further complicated by claims that any limitations on hate speech endanger people's right to freedom of expression. Despite the ambiguity of and political debates surrounding the term itself, hate speech has also been discussed as a technological problem: on the one hand, it is a problem because social media platforms and their algorithms help generate hateful and intolerant communication and its wide reach in society (e.g., Sharma, 2013; Massanari, 2015; Matamoros-Fernández, 2017; Udupa and Pohjonen, 2019), while on the other machine learning developers and researchers find it challenging to identify and thus monitor hateful content online (e.g., Burnap and Williams, 2015). Various algorithmic solutions for hate speech recognition and prevention are being developed both by platform companies and as part of academic research projects. Most of the existing solutions, however, are proprietary systems whose functionalities and performance are not publicly disclosed—as platform users, we only live with the deliverables and the decisions produced by these systems (e.g., Brown, 2018; Bucher, 2018).

In this paper, we reflect on a collaborative project in which a hate speech detection system was developed and implemented and discuss the expectations and responses elicited by the machine learning system from a critical data and algorithm studies perspective (Boyd and Crawford, 2012; Gillespie, 2014; Kitchin, 2014; Iliadis and Russo, 2016). For the project, the social media activity of candidates was monitored for potential hate speech during municipal election campaigning in Finland in April 2017. The monitoring project was initiated by an NGO, and it involved another NGO, the Non-Discrimination Ombudsman (later NDO, a governmental body to prevent and monitor discrimination), a software company, and researchers from two universities. Those collaborating on the project designed technical infrastructures to automatically filter potential hate speech from large social media monitoring data and developed measures to react to detected hate speech, for which a separate taskforce and process at the NDO office was initiated. All political parties were informed that their activities would be monitored. During the project, the public social media messages of all candidates were collected from social media platform APIs, classified using a machine learning system created for the project, and sent to the NDO for manual checking and for potential follow-up procedures. Manually given scores were used to retrain the algorithmic model during the project.

While we also report on the process and results of developing the machine learning system to detect Finnish language hate speech, the main focus of this paper is to critically discuss the building of the system and the ways in which participants interacted with the implemented algorithm. The purpose of the paper is, hence, 2-fold: first, to report and reflect on the choices made during the intervention project in more detail than a regular scientific report on the implemented system would allow for, and second, to critically discuss the expectations and challenges related to machine learning technology and the process of datafying hate. Hence, the involvement of researchers in the monitoring project can be described as action research, wherein the goal was not only to observe the research subject but also to develop its activities by participating in the activities of the studied community (Kemmis and Wilkinson, 1998). By acting as team members in the project, we had an opportunity to follow the planning and development of the technical system as well as to observe the sensemaking related to algorithm-based solutions during the project organization phase. The reflections presented in this paper are based on observations and notes made by the researchers taking part in the project, artifacts designed and collected during the project, as well as interviews with the project participants. Based on this material, we ask the following research questions: How can algorithmic methods be used to detect a complex socio-linguistic issue, such as hate speech? What kinds of expectations and experiences arise in a multidisciplinary project team when engaging in datafying and quantifying hate speech?

The paper proceeds as follows. First, we discuss the existing literature and research on hate speech and existing computational approaches to detecting hate speech. Next, we present our case in more detail and describe the nature of our own involvement in the hate speech detection project as researchers. The analysis part of the paper presents and discusses the stages of the identification project and reflects on the issues and questions that arose while developing the model and working with social media data. We conclude by drawing together our observations from the project and by discussing the practical implications.

### Definitions and Research on Hate Speech

The phenomenon that we nowadays refer to as “hate speech” is an old one. On various occasions in history, offensive speech has been used to target individuals or groups with the purpose of stigmatization and to incite hatred and violence. Hate speech was used as a tool in, for example, the Nazi holocaust and in the genocide in Ruanda. Public concerns about hate speech grew especially after the events of the Second World War (Bleich, 2011), and in the light of such events, several international treaties were signed, most importantly the Universal Declaration of Human Rights (UDHR) (1948), and the International Covenant on Civil and Political Rights (ICCPR) (1966). These treaties now recognize the rights of each individual to, for example, equality, personal dignity, security, and freedom of opinion and expression (UDHR, articles 1–3, 19), and forbid all forms of discrimination in violation of those rights (UDHR, article 7). More specifically, the treaties forbid engaging in propaganda for purposes of war or appealing to national, racial, or religious prejudices to incite hostility, discrimination, or violence (ICCPR, article 20). Other treaties legislate against racial discrimination, genocide, and other violations of international law (Convention on the Prevention Punishment of the Crime of Genocide, 1948; International Convention on the Elimination of All Forms of Racial Discrimination (ICERD), 1965). The most severe forms of “hate speech” can be defined and recognized based on these international treaties.

In the European context, the debate over “hate speech” in the past few decades has revolved around questions of ethnicity, religion, multiculturalism, and nationalism. Several European countries now have large migrant minorities consisting of many ethnic and religious groups. While this type of multiculturalism is a living reality, it has also become a source of criticism; the most critical voices claim that the whole ideology of multiculturalism has failed (for more on this so-called multiculturalism backlash, see e.g., Vertovec and Wessendorf, 2010). Next to much genuine societal and political debate, Islamophobic, xenophobic, and nationalist views have also gained ground, claiming that especially the Muslim minorities constitute a danger to European societies and the Western lifestyle, which need to be protected from more such influences (Vertovec and Wessendorf, 2010). These fears have been echoed by several nationalist parties, such as Partij van de Vrijheid (PVV) in the Netherlands, Front National (FN) in France, Alternative für Deutschland (AfD) in Germany, or the Finnish Perussuomalaiset (the “Finns Party”). Islamophobic and xenophobic sentiments and related hate speech have become prominent in public debates, especially after ISIS-backed terroristic attacks in several European cities in 2015–2017 as well as during and after the European “refugee crisis” in 2015 (e.g., Berry et al., 2015; Baider et al., 2017). Hate speech targeting various ethnic, migrant, and religious minorities flourishes especially in general social media discussions (for the Finnish context, see Pöyhtäri et al., 2019), but it has also long taken the forms of organized propaganda, hate groups, and hate sites (Roversi, 2008; Daniels, 2009; Citron, 2014; Anat and Matamoros-Fernandez, 2016; Brown, 2018; Farkas and Neumayer, 2018).

Despite the ongoing heated public debate, legislation in most European countries, including Finland, does not contain a definition for any criminal act termed “hate speech.” The incentives of European countries to tackle and prevent illegal online content have been presented in various statements, such as the recommendation given by the European Commission in 2018 and the code of conduct on countering illegal online hate speech by the European Commission (2016), together with stances adopted by Facebook, Microsoft, Twitter, and YouTube. The most significant international document to date is the Rabat Action Plan (OHCHR, 2013), formulated in a series of workshops by the United Nations. The Finnish discussion on hate speech often refers to the Council of Europe's Committee of Ministers (1997) on hate speech, which defines it as follows: “Hate speech covers all forms of expression which spread, incite, promote or justify racial hatred, xenophobia, antisemitism or other forms of hatred based on intolerance.” Additionally, the Finnish criminal law code defines various offenses that possibly consist of or contain hate speech, such as incitement to hatred (Rikoslaki/Criminal Code 11§10)^1^, defamation (Criminal Code 24§9), or illegal assault (Criminal Code 25§7). In the past years, several cases dealing with hate speech were handled in the Finnish courts, cases that now form a legal praxis on hate speech in the Finnish context. In most of these cases, hate speech had been used to incite hatred against Muslims or immigrants on social media platforms (Sisäministeriö, 2019). The question is legally difficult, however, since court cases must fall under the scope of the above-mentioned codes. When they do, they could be considered as cases of *hard* or overt hate speech in contrast to *soft* or covert hate speech, following the terminology suggested by Baider et al. (2017), which is not illegal but still raises concerns regarding discrimination.

Even though hate speech can be defined based on international treaties and legislation, the situation is further complicated by the colloquial use of the term (cf. Brown, 2017a; Udupa and Pohjonen, 2019). “Hate speech” now refers to a variety of speech acts and other ill behavior both offline and online, ranging from the penal criminal acts discussed above to speech and behavior that is uncivil and disturbing, yet tolerated. Those advocating the most liberal viewpoints often claim that any restrictions on freedom of expression based on hate speech accusations severely violate the basic right of free speech (e.g., Molnar, 2012; Citron, 2014). Altogether, this complicates the everyday understanding of or chance to reach a general consensus on just what constitutes hate speech. In its most colloquial and broad-based definition, hate speech can refer to, for example, verbal discrimination or attacks against various non-ethnic minorities, political hate speech, misogyny, violent pornography, online bullying and harassment, trolling, or doxing—and it has also been referred to as, for example, cyberhate (Edelstein and Wolf, 2013; Brown, 2018), cyber violence (United Nations Broadband Commission, 2015), or toxic speech (e.g., Perspective API).

Indeed, one ongoing debate has to do with what potentially can be regarded as a speech act severe enough to actually constitute illegal hate speech, which groups should be protected from hate speech, and whether the harms caused by hate speech should be considered actual and direct or societal and indirect (e.g., Calvert, 1997; Article 19, 2015; Udupa and Pohjonen, 2019). These debates are reflected in the theoretical discussion on hate speech as discourse, a form of othering that does not necessitate that actual or overt hatred be expressed in words—a speech act or discourse can contain a covert expression of hatred, embedded in the context of the speech act (e.g., Brown, 2017a,b; Baider, 2019, forthcoming). Such discourses do not necessarily have concrete, real-life consequences; rather, they contribute to the overall atmosphere regarding, for example, minorities. They are discourses connected to positions of power; individuals are often the targets of hate as representatives of a minority group (e.g., Brown, 2017a). Similarly, research on online hate and racism has discussed the *covert* forms of hate speech, textual and platformed practices that are not necessarily direct expressions of hate but support the circulation of hate and are used to stir up hatred as well as support hate communities (Anat and Matamoros-Fernandez, 2016; Brown, 2018).

Hate speech or online hate is thus a complicated set of practices not easily reduced to mere content features of the speech act (see Brown, 2017a). While legal and discursive definitions exist, ongoing debates about those definitions as well as considerations related to the context and reach of the speech act still persist. Such contextual factors are included in some hate speech guidelines and materials, such as the Rabat Action Plan (OHCHR, 2013) and NGO-produced materials like the Article 19 Hate Speech Toolkit, as well as discussed widely in academic research (e.g., Baider et al., 2017; Brown, 2017a,b; Relia et al., 2019). In our project, we chose to build on a broader definition of hate speech than the one allowed for by Finnish legislation and aimed to cover the forms of speech that can be considered problematic as a discourse. These considerations will be explained in detail in section Defining and Quantifying Hate Speech.

### Algorithmic Approaches to Detecting Hate Speech

Despite the contested nature of the term, various online platforms have engaged in projects that aim to counter and detect hate speech in their content streams, mostly due to increased public pressure. Various platforms have communicated their good deeds and success with hate speech removal: for instance, Facebook tries to moderate hateful and discriminating content, disinformation, violent content, and certain harmful ideologies (Koebler and Cox, 2018; Roberts, 2019; Sandberg, 2019). The platform has also reported building systems to identify hate speech based on images, such as memes (Sivakumar, 2018). YouTube has reported removing millions of videos with violent or extremist content (Hern, 2018) and shut down channels of far-right actors (e.g., Alexander, 2019). In the summer of 2019, YouTube announced an update to its policies prohibiting hateful content, such as violent extremism, Nazi ideology, and supremacism (YouTube, 2019). These actions are not only examples of corporate goodwill, but also forms of soft regulation and reactions to regulations given by governmental actors (e.g., European Commission, 2016, 2018).

With increased public discussion on hate speech, academics have attempted to create models that can automatically identify hate speech. Most of these approaches rely on word lists, bag-of-word approaches, or ngrams (e.g., Greevy and Smeaton, 2004; Pendar, 2007; Chen et al., 2012). Some more recent detectors utilize bag-of-word vectors combined with word dependencies to identify syntactic grammatical relationships in a sentence (Burnap and Williams, 2015), semantic word embeddings (Badjatiya et al., 2017), or neural networks (Al-Makhadmeh and Tolba, 2019; Relia et al., 2019). Many of these studies highlight the difficulties inherent in such a process, particularly the problem of separating hate speech from other types of offensive language (e.g., Davidson et al., 2017). Methods that somehow take into account word contexts are important: consider, for example, the sentence “Send them all back home.” It indicates a covert form of hate speech: none of the words as such are indicative of hate, but the combination of words generates a call to action, particularly when discussed in the context of immigrants. Further, all these models are dependent on training data annotated by humans, which is a laborious process that involves potential biases. Waseem (2016), for example, showed that amateur annotators are more eager to label messages as hate speech than trained annotators.

Hate speech detection systems, particularly the ones in industrial use, have been criticized for their inadequacy and inconsistencies (e.g., Makuch and Lamoureux, 2019; Sankin, 2019), and it is easy to find examples of content that has gone undetected and yet clearly should be prohibited according to existing content policies. Further, Viejo (2017) has criticized the individualistic nature of hate speech moderation by the various platforms: instead of considering hate speech as a historical, societal entity, it is a matter of individuals being hurt, silenced, or banned. Furthermore, their functionality is difficult to evaluate since the algorithms are corporate black boxes whose principles, parameters, or even the methods used are not transparent (e.g., Brown, 2018; Bucher, 2018). Despite these problems, the level of trust in using algorithms and automation to solve the problem of hate speech seems strong. While algorithms can be used to handle large amounts of data and make decisions faster and more efficiently than humans could, they also hold rhetorical power, as Gillespie (2016, p. 23–24) states: “Conclusions described as having been generated by an algorithm wear a powerful legitimacy, much the way statistical data bolster scientific claims. It is a different kind of legitimacy from one that rests on the subjective expertise of an editor or a consultant, though it is important not to assume that it trumps such claims in all cases.” Algorithms, thus, hold social power through the ideas and notions attached to them in social contexts (Beer, 2016).

These discourses and forms of power are not meaningless. Science and technology studies have highlighted the generative role of future expectations and orientations in technology development: the interest in new capabilities and possibilities also works to mobilize both social action and economic resources (Borup et al., 2006; Beckert, 2016). Our expectations in relation to algorithms, algorithmic systems, and machine learning thus mediate the future-oriented decisions we make, promote certain kinds of decisions, and rule out others (Mackenzie, 2017). This is why critical studies of algorithmic systems and the contexts in which they are designed, programed, and implemented are of paramount importance.

## Case, Materials, and Methods

This study builds on observations made during a hate speech identification project conducted in Finland for the municipal elections in spring 2017. Our approach is a combination of ethnographic observations and action research, where the goal was not only to observe the research subject but also to develop its activities by participating in the activities of the studied community (Kemmis and Wilkinson, 1998). The project was a pilot project on hate speech identification in Finnish social media initiated by two NGOs, the governmental office of the Non-Discrimination Ombudsman (NDO), and one software company. Researchers representing two universities and three academic research projects related to the topical area were invited to join the project at a later stage. We took part in only one planning meeting, then joined the team to advise and help them in designing the hate speech detection model, were involved in a hands-on manner in the monitoring phase, and joined the debriefing meeting. Thus, as researchers we were actively involved in the project, made decisions concerning the project, and were also in a unique position to follow the activities and sensemaking of other participants. Further, our data includes the developed model itself, the datasets constructed during the project, internal social media discussions and informal meeting notes made during the project, and a collection of media coverage and social media commentaries elicited by the project. The material allowed us to follow the project through its entire lifetime, from the planning stage to reflections afterwards.

In combination with participatory action research, we used ethnographic methods to engage in sensemaking with the participants and recognize the ways in which their interpretations are socially constructed in connection the technological aspects of the project (e.g., Allard-Poesi, 2005). Instead of committing ourselves to certain theories or general hypotheses, we approached the studied phenomena openly, becoming sensitized to its features. For this purpose, we applied the ethnographic premise of carefully following what happened around us, listening to different stakeholders, and asking questions and collecting artifacts, such as news articles, social media data, and internal communications (chat platform, emails), in conjunction with personal notes (for more about ethnographic methods, see, e.g., Hammerslay and Atkinson, 1995; Gobo, 2008). In addition, in an action-research-oriented setting, such as ours the researchers themselves are participants and their own actions and interpretations also become part of the research material.

Finally, to obtain a better picture of the views of the organizations involved, interviews were conducted with the NGO and NDO representatives after the project had concluded. Once the project was officially finished, regular contact among the participants ended too, and ethnographic fieldwork thus became an unviable data gathering method. One of the authors, who was not involved in the project itself, interviewed three participants, each from one non-university organization in the project. The themes of the interviews included, for example, participants' own reflections on the project, their expectations for the model, and how they felt about the model that was created. One of the interviewees was the person who initiated the collaboration. The interviews took ~1 h each, they were conducted in Finnish, and transcribed verbatim. For the purposes of this study, the interview data was initially analyzed using an open coding scheme to systematically map out different themes that the interviewees discussed and to become familiar with the data. The two first authors then discussed the overall focus of inquiry and the gaps that the ethnographic approach had not been able to fill and went back to the interviews once again to identify answers to such questions as how the participants felt about the project's achievements afterwards and reflect on how they matched their initial expectations. The interviews were also used to gather additional information on the motivations of the organizations to participate in the project. Thus, the interview material used in this study fell mostly under the themes of “motivations,” “hopes and expectations,” and “consequences.”

Furthermore, as part of the project we also investigated a large amount of empirical data collected from various social media platforms. The nature and procedures done with this dataset will be explained in detail below in the analysis section when we describe the course of the project and reflect on the choices and decisions made during the development of the machine learning model. By doing this, we aim to shed light on the more detailed questions and problems that arise during any research project or industry project that works to develop an algorithmic system.

Neither an ethical review nor prior approval are required for a study on human participants in accordance with the local legislation and authors' institutional requirements. All participants gave written informed consent in accordance with *The ethical principles of research with human participants and ethical review in the human sciences* in Finland given by the Finnish National Board on Research Integrity TENK. However, for research ethical reasons all the excerpts from the social media data presented in this article are shown without reference to the source of the message and have been translated from Finnish into English to prevent their further spread and tracking (Kosonen et al., 2018). However, since hate speech is something heavily related to linguistic forms, we chose not to alter the content more (on fabrication, see Markham, 2012). Further, we also opted not to mark the source organization behind the interview quotes since only three organizations were interviewed and due to the public nature of the project, which would have made them easy to identify.

## Results and Reflections

### Intentions, Hopes, and Expectations

In the interviews, we asked the participants to reflect on their intentions and expectations when joining the project. One NGO acted as the recruiting and initiating actor for the project, but for all participants the project theme was somehow related to their otherwise ongoing work. The interviewee from the initiating NGO stated that he had a personal interest in democracy and elections in addition to interests related to his work, which included monitoring election and candidate data. The idea of potentially being able to automatically detect hate speech had, according to the interviewee, formed over time as a result of interactions with different people. At some point, he started asking around if other organizations would be interested in attempting something more concrete in the field. This outreach led to the initiation of the monitoring project. The interviewee also stated that he considered the municipal elections a good venue for testing the model, since they generally include a large number of candidates who are not necessarily professional politicians, meaning that the language they use might differ from those who are more experienced in expressing themselves publicly. Since the project did not have specific funding, the interviewee also described it as “goodwill” from their organization and from the software company, which has its own program for focusing efforts on societal projects. Another NGO and the governmental body saw the project as a small amount of extra work that related to their ongoing work against discrimination. Both organizations had encouraged all Finnish political parties to a sign the Charter of European Political Parties for a Non-Racist Society in 2015, 2008, and 2003. As such, they were willing to try a new approach to the topic. For the governmental body, it was “a self-evident” effort to counter hate speech precisely because the Finnish law states that the body's mission is to counter discrimination, and they saw hate speech as one form of discrimination.

One participant from an NGO stated that they “wanted to learn about the ‘thoughts' of machines,” meaning that their organization saw this project as a learning opportunity given that in the current societal situation, different automated technologies have started to be applied in new areas. While this interviewee was critical of claims, or of proving claims, that hate speech has increased because of social media, the participant did state that social media has fostered new forms of hate speech. These forms could then be approached with new kinds of methods. In the first project meetings, hopes were placed in the system to work as an all-seeing eye that finds all corners of the net and shines a light on hate speech that would not be noticed otherwise (see MacCormick, 2012), and also that the system would function as a mechanism to promote the use of respectful language by political candidates.

Hence, the beginning of the project was driven not only by an acknowledgment of the issue of hate speech as a societal problem, but also by the magic, myths, and drama present in the societal discourses regarding big data, automated intelligence, and algorithms (e.g., Gillespie, 2016; Ziewitz, 2016). We must acknowledge this was also an initial point of interest for us researchers to become involved with the project: to engage in an opportunity for new method development, but also with hopes to bring scientific rigor to the design and implementation of a model to monitor hate speech.

### Everything Starts From the Training Data

Before a supervised machine learning algorithm becomes useful, it needs a set of data that can be used to train the “ground truth” for the classifier. It is well-known that the quality and content of the training data highly affects machine learning algorithms (e.g., Friedman et al., 2001; Mackenzie, 2017). Therefore, by choosing the dataset we at the same time gave additional cues to the machine learning model as to what kind of hate speech we were looking for. The biases potentially caused by the training data are sometimes rather obvious in existing systems. For example, the toxicity scores given by the Google Jigsaw Conversation AI, a state-of-the-art model for toxic language detection, have been accused of giving higher toxicity scores to sentences that include female/women than male/men (Jigsaw, 2018). Such differences are due to the over-representation of certain classes in the training data that the system is built on: unless carefully balanced, any collected real-life dataset contains more toxic comments concerning women, so the evaluation of toxicity becomes attached to those specific words that should only be the “neutral context.”

Being aware of this limitation, we tried to create a training dataset that was as balanced as possible. First, we used a subset of a dataset of Facebook discussions from politically-inclined, Finnish-language public Facebook groups collected for another, racism-related research project the authors were involved in as well as another dataset containing messages concerning political groups from the largest Finnish-language online discussion board (Lagus et al., 2016). The Facebook messages was queried from the larger database using a list of words related to minorities. The word list was taken directly from a report on hate crimes by the Finnish Police University College, where the same words were used to query hate crimes from their internal database of police reports (Rauta, 2017). For us researchers, this was a common, acceptable strategy of externalizing some choices made in the research done for existing studies or investigations, and thus a way to circumvent some of the definitional difficulties related to the concept of hate speech.

Since the most common form of hate speech identified in Finnish society is related to ethnic minorities (Rauta, 2017, 2019), our dataset of racism-related online discussions could be considered feasible. However, we were aware of possible criticisms that could be raised by the project, like accusations of censorship for certain types/topics of speech, and therefore we specifically aimed to include data that also targeted other minorities, such as the disabled or the Swedish-speaking minority. In addition, we anticipated that such a monitoring project would encounter criticisms for only focusing on minorities and dismissing hate speech targeted at politicians. Therefore, we extended our original training dataset with online discussion board data filtered with pejorative words related to named political groups: a word that referred to the True Finns Party (“perpanssi,” 50 messages) and a word that is the Finnish equivalent for a social justice warrior (“suvakki,” 50 messages).

### Defining and Quantifying Hate Speech

Since neither the academic community nor Finnish law offer a clear definition of hate speech, we had to start by formulating our own definition for the data classification purposes. As discussed in section Definitions and Research on Hate Speech, we aimed to cover both illegal and “legal” forms of hate speech, while leaving the final judgment to the NDO lawyers. We grounded our definition in the Council of Europe's Committee of Ministers Recommendation 97(20) definition of hate speech: “Hate speech covers all forms of expression which spread, incite, promote or justify racial hatred, xenophobia, antisemitism or other forms of hatred based on intolerance.” Further, we used the materials compiled by the NGO Article 19 (2015) and their six-part test for hate speech identification as well as materials produced by the Ethical Journalism Network^2^ for journalists to identify hate speech, which builds on the principles postulated in the Rabat Action Plan (OHCHR, 2013). The latter two groups of material strongly advocate a focus that looks beyond the content of the speech and takes into account the context, the status of the speaker, and the potential consequences of the speech act. We discussed these aspects during our planning stage but acknowledged that they would be difficult to reach with our computational approach.

Some of the contextual elements are easier to control for: for example, in our context, marked by elections, the status of the speaker was clear; each person followed was a candidate and thus speaking from a somewhat significant political position legitimized by the party. The questions related to context and the potential harm incited by hate speech are much more complex. We considered different ways to examine the context of the expression, including for example downloading the message thread in which the original message was posted or running some analyses on the posters' accounts, as suggested by ElSherief et al. (2018). However, expanding our data collection to include the context or the profiles would also mean including messages from non-political actors, such as ordinary citizens, in our data collection. Such surveillance would require solid justifications—particularly if done by a project that includes a governmental actor. We opted, thus, to leave the evaluation of the context and the potential consequences to the manual checking phase done by the NDO representatives after the potential hate speech messages had been identified by the system.

Further, we aimed to include some of the more fine-grained definitions in our code book in order to reveal legal but problematic forms of hate speech discourse. In addition to annotating the level of hate speech, we used the above-mentioned definitions and tests to generate the following list of features characteristic of a message in the clearly denoted hate speech category: the message contains (1) a call to violent action; (2) a call to discriminate or to promote discrimination; (3) an attempt to degrade human dignity based on their characteristics; (4) a threat of violence or the promotion of violent action; or (5) contempt, solicitation, name calling, or slandering. Originally, we planned to annotate the presence of these features in the messages. In addition, our original classification plan included fourteen different labels used to identify the group toward which the speech is directed. However, in the end we never used these more specific labels beyond the severity level of the speech, as classifying even that level of speech proved to be more difficult and time consuming than expected. Such a multi-level classification system has, however, been successfully presented in some recent academic publications (Burnap and Williams, 2016; Relia et al., 2019).

When annotating the severity of hate speech, we used a scale that ranged from 0 to 3 (with 3 clearly indicating hate speech, 2 indicating disturbing angry speech, 1 indicating normal discussion with a critical tone, and 0 being neutral). While hate speech, particularly in a legal sense, is a binary classification task, we opted to use a more nuanced scale in order to better explore the phenomena and its severity in the Finnish context and to generate a dataset that could potentially be used for other purposes later on. It took us an extensive amount of time and codebook refining to reach a reasonable level of consensus among the annotators. With four coders—two of the authors, one NGO representative and a research assistant, none of whom could at that point be regarded as an expert on hate speech—we spent almost 6 h coding subsets of 100 messages before reaching an acceptable level of agreement, as measured by Cohen's kappa (>0.7), while discussing our classification principles after each failed round. After that, the rest of the training set was coded by four researchers individually.

ALL SOMALIS SHOULD BE PUT TO CATTLE CAR AND SENT TO THE DESERT VIA SIPERIA, STIFFED…

[training data, annotated level 3]

*If a native Finn complains about a decision made by an official, like a parking fee, you'll get another fee for 250 euros. I guess there is a fee for immigrants as well, right??*
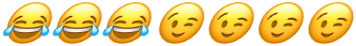

[training data, annotated level 2, in a discussion on asylum seekers' appealing negative decisions regarding their refugee status]

As implied by the examples above, it must be acknowledged that the variety of messages was so broad that we probably could still find messages on which we disagree. After each round of coding, we discussed each example on which we disagreed and worked to build a joint conception of the features of hate speech. It became clear that the coder's own knowledge of the issue and related expressions affected their judgments. For example, a person might easily recognize particular slur words if previously encountered. During our classification, we discussed, for instance, the expression “John of the night” (“yön Timo” in Finnish), a pejorative word used to refer to colored people in Finland. One of our annotators had never encountered the term before, and the hateful content of the message was not obvious without this prior knowledge: the message seemed to be about a specific person instead of referring to an entire group of people with a group noun. Contrasting observations are discussed by Waseem (2016), who showed that amateur coders are more likely to classify content as hate speech than trained experts. Likewise, Davidson et al. (2017) highlighted the cultural connotations at play, as they found that messages with racist or homophobic content were more likely to be classified as hate speech than sexist messages, which were generally classified only as offensive.

The target of the speech, however, does affect the severity of the case, as postulated by the Finnish law. The Criminal Code (24§9) condemns defamation but makes an exemption for critiques targeted at a person's actions in politics, business, or another public position. Also, several of the hate speech definitions emphasize discrimination and minorities, which means that hate speech is more severe when targeting a minority instead of the majority, such as white heterosexual men in the case of Finland. For these reasons, in the example message included below the first part of the criticism, directed at a politician, is not considered hate speech, but the latter part, which denounces all people living in the countryside, could be considered hate speech:

“*[a populist politician] is a complicated case because he is [like] a bunch of dicks. After hearing his babble about the refugees, we know his stance on nature protection, ladies, gays, large carnivores, peat, war, dissidents, raping the forests. No matter what the case, opinions like his are at least for me extremely obnoxious. Btw, in the countryside 90 percent of the population are similar dick-bunch people. Regardless of the gender.”*

[training data, annotated level 3]

### Development of the Machine Learning System

To accomplish the project goals, we developed a tool that processes messages in social media and highlights the most likely messages containing hate speech for manual inspection. While other studies have created hate speech identification systems, industry solutions, and existing libraries, none of them could be directly used for the process, as they typically were built and trained for English-language data. Our project was, to our knowledge, the first hate speech detection model done in Finland, apart from proprietary machine learning systems that exist in companies that offer automated moderation tools for media organizations. Therefore, we built and tested a custom text classification model. Using standard libraries, we tested different machine learning algorithms to identify the one that would perform the best.

The text data was preprocessed and cleaned using standard text mining approaches, most notably word stemming. To train such a model, our original four-level scale was reduced to a binary classification of clearly denoted hate speech vs. other types of speech. The dataset was rather skewed even with the four-level scale, with non-hate speech dominating the dataset. In addition to the binary classification, our algorithm gave a probability score for each message, which was then used to sort messages based on how likely they were to contain hate speech. Hence, by following the necessities of the selected approach the textual training data was quantified and abstracted to a format that allowed for the transformation of hate into probabilities (see Mackenzie, 2013).

**Figure 1:**
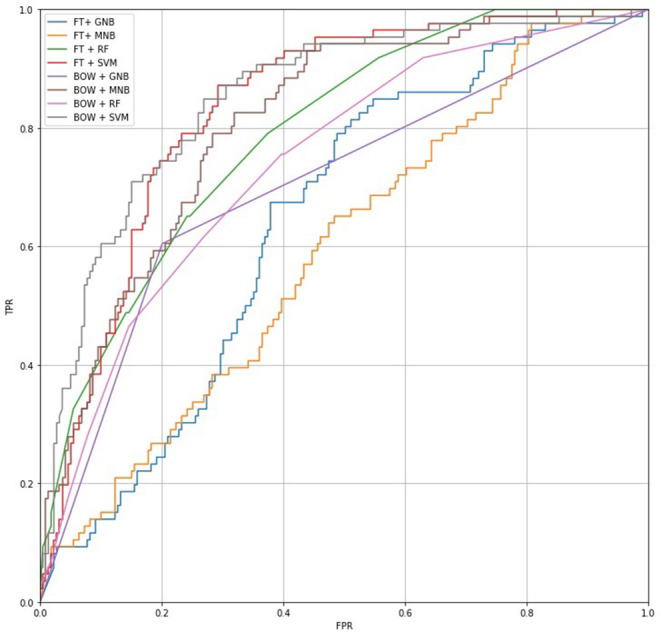
ROC curves mapping True Positive Rate (TPR) and False Positive Rate (FRP) for each feature extraction/machine learning method combination.

The training set was used to select a feature extraction and machine learning method and to train a model for hate speech detection (Friedman et al., 2001). We followed the best practices in applied machine learning and divided the collected dataset into a training dataset (90%) and a test dataset (10%). The training dataset was used to train the model, while the test dataset was used only to evaluate the performance. Further, since no existing implementations for detecting hate speech in Finnish exist, we tested and compared the performance of different method combinations: TF-IDF weighted Bag-of-Words (BOW) (Sparck, 1972) and FastText with pre-trained Finnish word embeddings (FT) (Bojanowski et al., 2017) for feature extraction, and Gaussian Naive Bayes (GNB), Multinomial Naive Bayes (MNB), Random Forest (RF), and Support Vector Machines (SVM) as machine learning methods. This set of methods was chosen because the methods have been widely applied in text classification tasks and other challenging machine learning tasks, such as spam filtering or document organization (e.g., Aggarwal and Zhai, 2012). The results of the experiment are depicted as the receiver operating characteristic curve in Figure 1; precision, recall, and the F1-score are presented in Table 1.

**Table 1:** Table of test results for different metrics for each feature extraction/machine learning method combination with threshold = 0.5.

**Method**	**Detecting non-hate speech messages**			**Detecting hate speech messages**				**Accuracy**
	**Precision**	**Recall**	**F1-score**	**Precision**	**Recall**	**F1-score**	**ROC AUC**	
FT + GNB	0.731034	0.968037	0.833006	0.533333	0.093023	0.158416	0.642588	0.721311
FT + MNB	0.732283	0.849315	0.786469	0.352941	0.209302	0.262774	0.592837	0.668852
FT + RF	0.781132	0.945205	0.855372	0.700000	0.325581	0.444444	0.788999	0.770492
FT + SVM	0.806723	0.876712	0.840263	0.597015	0.465116	0.522876	0.833333	0.760656
BOW + GNB	0.837321	0.799087	0.817757	0.541667	0.604651	0.571429	0.701869	0.744262
BOW + MNB	0.718033	1.000000	0.835878	0.000000	0.000000	0.000000	0.808219	0.718033
BOW + RF	0.746429	0.954338	0.837675	0.600000	0.174419	0.270270	0.740708	0.734426
BOW + SVM	0.782288	0.968037	0.865306	0.794118	0.313953	0.450000	0.851572	0.783607

The BOW + SVM combination achieved the best performance in the experiment, clearly outperforming other methods and providing a recall of 0.3140, the highest level of precision (0.7941), and the highest ROC AUC (0.8516). The ROC (receiver operating characteristic curve) measure in particular was important for us because we wanted to sort the messages based on the likelihood they could be classified hate speech and did not want to miss any hateful messages. Figure 1 shows the ratio between the True Positive Rate (TPR) and False Positive Rate (FPR): the FPR axis describes the mistake ratio (lower is better), while the TPR axis describes the overall success rate (higher is better). The challenge is to find a balance such that the TPR is high but the FPR low. Based on the results, we chose to use a combination of BOW and SVM to detect hate speech.

### Ready, Set, Go! Monitoring Phase and Project Results

The streaming data collection acquired during the monitoring period was limited to politicians who had signed up as candidates in any Finnish municipality and who were publicly campaigning on Facebook or Twitter. The Twitter handles or Facebook page URLs were extracted from voting advice application data published by the national broadcasting company YLE—the cleaning of this data also took several working days. While over 33,000 candidates signed up for the elections nationwide, only a limited number of them used social media for public campaigning: our final streaming data collection included 6,400 Facebook pages and 1,308 Twitter profiles, which altogether produced 26,618 post for inspection. The data sheet contained the message id, timestamp, post-author, post-content, context platform, and the original url. While planning the data collection, we also reflected on the legality of the project and read through the Finnish Personal Data Act, concluding that it gives permission to collect data from persons in a public position (Personal Data Act, 1999).

**Figure 2:**
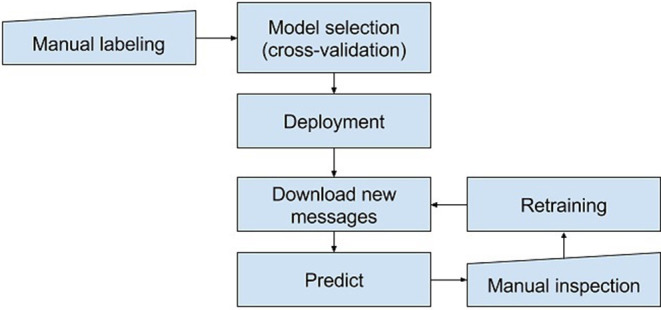
The process model of the hate speech detection.

During the month prior to election day, we ran the detector once a day. We downloaded social media messages posted on Facebook or Twitter by the candidates during the previous day, then predicted the level of hate speech (i.e., scored each message using the trained model) and stored the results in a CSV file. The CSV contained only the messages and prediction scores; no usernames or URLs were included. The CSV was sent for manual inspection to the NDO representatives, who were instructed to sort the messages by the prediction score and label them using the same four-level annotation scale used for the training data. As explained above, this step constituted the final qualification as to whether the identified speech act was illegal hate speech or hate speech according to the Council of Europe's Committee of Ministers (1997). After the manual screening phase, the generated new samples were used to retrain the hate speech detection model (see Figure 2). The government officers working at the NDO office are trained lawyers, whose work focuses on the issue of discrimination. Therefore, we agreed they would be the ones providing the final “ground truth,” the experts with the final word regarding the level of hate speech found in each message.

During the monitoring project, we found that the model gave rather high scores for messages not containing hate speech when compared to the results of the manual inspection. This might be due to the fact that the detection algorithm was originally trained with a more biased set of samples compared to the actual incidence of hate speech in online political speech—or at least in candidate messages—or compared to a stricter classification done by the training data annotators. Another reason might be that the training dataset did not consist of messages written by politicians, but those written by regular citizens, which might imply a different language style—this is a clear limitation of our machine learning model. The model predictions, however, were improved by retraining it during the monitoring phase. In the end, only 205 out of a total dataset of 26,618 posts were classified as hate speech by the machine learning system. After the manual screening, it was determined that only five posts contained hate speech that required further measures, while 43 messages almost constituted hate speech. In the end, the NDO representative filed charges with the police for one post, but this message was identified manually and not by the machine learning system. Two notification letters were sent to parties concerning their candidates' messages.

After the project, the source code of the created model was published as an open source with an MIT license on GitHub (Futurice, 2017). This was an act required by the policies of the participating software company and also an act of algorithmic transparency advocated by the project team. The open availability of the algorithm, however, does not reveal much about the system without the training data used to train the algorithm. We decided not to publish the data for two reasons: first, releasing a set of potentially toxic messages would only make such discourses more widespread, and second, the publicizing of social media datasets is restricted by the major platforms (see Williams et al., 2017).

### Participants' Views After the Project

Our interviews show that in the end, the participating organizations did not receive direct, measurable gains from the project, which might reflect the original, perhaps too grandiose, expectations. One of the NGO representatives stated that their vision seemed a bit naive in hindsight: “I had a vision that there might be some kind of dashboard where you could see the amount of hate speech or the level of anger in that speech.” The developed technology did not contain this kind of UI. Instead, the messages that were tagged as potential hate speech were sent to the governmental body as spreadsheets. The high-tech vision was then quite different from how information was conveyed from the system to those who used the information to make decisions. In a similar vein, the interviewee from the governmental body stated that the model still required too much manual work from their organization. While the intention of the researchers was to build a more nuanced classification scheme and, consequently, an algorithm that could detect these nuances, from the interviewee's perspective the model labeled too many messages as potentially hate speech, even with the truncated classification system:

“*well, if you ask me, like, if it worked, well it didn't {laughs}—or yes, it found too much stuff or basically too little; then it found too little, ehm, like something that one would have categorized strongly as hate speech, which led to us having to browse through a lot more messages, because, like, you couldn't trust it, it couldn't be trusted in any way.”*

However, the project was an important learning experience for the organizations, as they got to experience firsthand the requirements for creating a machine learning model and the human work needed. This also helped them to understand what kind of roles different experts could have in building such models. For example, one of the organizations mentioned the skill of asking the right questions from the data in order to build a better system—a notion also highlighted by critical data studies (e.g., Boyd and Crawford, 2012):

“*So, there should have been even more fundamental info given to [the annotators]. So that they won't start with a vague intuition, like this, that I feel that in the beginning it was not clear to them that, like, that not all angry speech is criminal—or, like, is clearly a criminal act. So, like, the basic question itself. But something interesting, yes, and at least I take the attitude that there we learned, right now, like, for example, how clearly one should pick their words, so that projects like that can be reasonably valuable.”*

Thus, the NGOs did consider the project useful in the larger sense: the project was seen as a small-scale pilot, an attempt to understand what could be achieved with advanced algorithmic methods. In fact, on some occasions, participating NGOs hesitated to even call it “a project” since it had no specific funding allocated to it and was more of an extra exercise related to the ongoing projects and interests of the participating organizations. This somewhat lean approach also caused some minor issues during the project when recent information was not always clearly communicated. For example, after the researchers were invited to join the project the governmental body expressed surprise that some new people began commenting on online documents about the project. For the NDO, it was important to know who was participating because they have a role as a public actor, which means that they have certain juridical responsibilities. For example, it was of utmost importance for them that the Finnish law should be followed.

Finally, the project elicited some unintended—if not unanticipated—consequences. The project received extensive coverage in the news media after a press release was sent by Aalto University. Consequently, the project participants received much direct and indirect criticism and even death threats. This included the researchers, but particularly the NDO representatives, who, as governmental actors, were seen to be exceeding their authority. For instance, the online newspaper of the True Finns Party covered the project with the headline “The Ministry of Justice was involved in building a system that hankered after each social media message by the candidates” (Suomen Uutiset, 2017). An official interrogatory to the Minister of Justice was submitted by the same party member concerning the actions of the NDO in the project. Further, public online discussion concerning the project in various online arenas (particularly the ones known to support populist views) described it as an illegal surveillance system that ran counter to freedom of speech, something that breaks the law and merely consumes the resources of society. Other critics suspected that the developed algorithm aligned with the political opinions of the actors developing it and had only been developed for political purposes and to exercise political power over anti-immigration groups. Online, the project was frequently referred to with dystopic accusations of acting as “the thought police” in an Orwellian society or likened to the Nazi Reich and East German Stasi. The online discussants were not fooled by the idea of objectivity often connected to algorithms (e.g., Beer, 2016; Markham et al., 2018): according to one anonymous forum poster, “algorithm is just a fancier name for a list of things that represents hate speech according to the one who made it. So, it's more like an opinion algorithm.” A few individuals also contacted the software company and requested to see the algorithms and the data.

The project team made the decision not to publish any of the monitored messages or to pinpoint the actors in any way so as not to give more publicity to the messages. Further, we did not want the main tone of the project to be incriminating, but rather to promote tolerant language use in political campaigning. In hindsight, we could ask if this was a mission accomplished in the sense that we indeed developed a hate speech monitoring tool, but the most visible consequence of the tool was that it ended up generating more hate speech in society through online discussions and directly targeting the NDO.

## Discussion

The collaborative project investigated in this study offered a unique viewpoint on hate speech detection from a dual perspective: how hate speech emerges as a technical problem, and how participants make sense of it while working with an algorithmic system. From a critical technology studies perspective, we acknowledge the problematic nature of datafying and quantifying (*cf*. van Dijck, 2014; Mackenzie, 2017) emotions and emotional language, such as hate speech. Recognizing hate speech is not an unambiguous task even for humans, which makes it a rather complicated task for machines—or a task that can be achieved in the sense that probabilities are given, but their validity must be critically evaluated. As discussed in the introduction and in previous studies on the phenomena (e.g., Baider et al., 2017; Brown, 2017a, 2018; Udupa and Pohjonen, 2019), hate speech is a concept with varying definitions, juridical interpretations, and cultural connotations, which makes the automated recognition of it a challenging technical endeavor—but precisely because of that, it represents a type of societal issue many actors are hoping to solve with technology. Next, we will discuss the problematic aspects of datafying hate by reflecting on our own experiences in the project.

First, the main goal of the project essentially turned out to be the quantification of hate as a single digit and a figure of anticipation (see Mackenzie, 2013). This happens, first, when classifying the training data, and second, when vectorizing the textual data for the machine learning method (Mackenzie, 2017). While an adequately well-working machine learning solution was developed in the project, the automated approach requires heavy simplification, such as using rudimentary scales for classifying hate speech, which in reality has several different tones and varieties. Indeed, hate speech is an evolving linguistic phenomenon, and its characteristics follow the discussions and trends in a given cultural context and in society at large. It is also a phenomenon constantly affected by the algorithmic systems on which our public communication takes place (*cf*. Sharma, 2013; Udupa and Pohjonen, 2019). Users as well are aware of the quantification and monitoring of specific keywords done by the social media platforms (e.g., Gerrard, 2018). That is why they constantly develop new ways of expressing such emotions as hate and intolerance more covertly, by, for example, misspelling words on purpose or generating new pejoratives or creative metaphors (cf. Baider et al., 2017; Brown, 2018). Think of, for instance, a rather offensive but cunningly masked statement given by a Finnish politician: “*An immigrant is a blemish on the street*.” Annotating the training data taught us that identifying hate speech is not clear even for humans; we had trouble reaching agreement, and we were forced to revisit the definitions several times before reaching a common understanding. In the process of conducting quantification and vectorization, we inevitably flatten the data and lose variety of expressions. This, however, is precisely what makes algorithms powerful through their ability of performing abstraction (Pasquinelli, 2015, cited in Mackenzie, 2017, p. 9).

Second, the existing methods of machine learning heavily build on existing vocabulary or lists of words, including the bag-of-words models and support vector machines used in this project. Hate speech, however, is not a phenomenon consisting only of words or lists of words, even though they can be indicative of hate (cf. Burnap and Williams, 2015; Udupa and Pohjonen, 2019). The actual sentiment or affective tone of a particular message relies immensely on the final form of the expression. While word vector models are somewhat sensitive to word contexts, when combined with the BOW approach they emphasize specific words as features when deploying the model. For some reason, the performance metrics for BOW seem to generally produce better results than FastText word embeddings, which should be more sensitive to word contexts and combinations.

The word-centered approach becomes even more problematic when working with social media data, which is quite specific by nature. It is characterized by vernacular expressions and contains mundane words and grammatical variance—which is particularly the case with the Finnish language, where the spoken and written language differ considerably. In addition, the forms of social media constantly develop to include more visual forms of communication. Not only are several platforms built around images and videos, but also the use of visual elements, such as emojis and gifs, is becoming more common on every platform. When treating social media data as text, these visual messages merely appear empty spaces. Take the visual forms of communication adequately into account would require more sophisticated data collection methods and, in practice, separate algorithms to identify any content from the visual messages. Identifying the sentiments underlying images or multimodal data is a task far more difficult than text-based sentiment analysis (e.g., Soleymania et al., 2017).

In this sense, following the standard state-of-the-art procedures in machine learning lead to a technical solution that is counter-intuitive to everything we know about hate speech as a social and contextual phenomenon and that is also highlighted in hate speech prevention recommendations, such as the Rabat Action Plan (OHCHR, 2013). In a project as technological as ours, it is intriguing to observe how we ended up developing and implementing solutions that placed faith in an algorithm, even though as social scientists we know the importance of contextual cues, such as message topic and the position of the speaker. Limited resources and the requirements of the process itself to deploy at least a relatively working system meant that much of what was done was dictated by the technology and expectations placed on it rather than the messiness of the empirical world. The magic of machine learning is that it is easy for actors to follow the programmatic knowledge production practices of the field, not only to organize data, but also to organize the relationships between humans and machines in the process (Mackenzie, 2017).

Apart from the lessons learned in the machine learning part of the study, the action-research-oriented setting allowed us to observe such affective responses as hopes, dreams, and fears related to the machine learning technology. Based on our observations, interviews with the project participants, and an analysis of the online discussions in reaction to the project, we identified how participants—even ourselves—had aspirations for effective automation as well as neutrality and objectivity introduced by the algorithm. In particular, the non-technical participants saw the system as a more objective mediating agent than a human actor because the identification of hate could be outsourced: one could call this the participants' imaginary (Bucher, 2017) for the machine learning algorithm, which changed during and after the project. This was prominent especially in the planning phase: with no deeper understanding of the algorithmic implementation behind the system, the participants had no expertise for questioning the functioning of the system before they were confronted with the automated classification proposed by the model. As described in this paper, the participants were ultimately disappointed with the boring, ordinary tools, such as the excel files used.

As pointed out by the interviewees in retrospect, the extra manual work needed to check the results, as well as the frequent errors made in the predictions, reduced the usability of the implemented system. The model was designed, tested and built following state-of-the-art computer science practices, but in the end, it was not capable of performing the task. The frustration of the participants reflects the general expectations of automation and efficiency related to algorithmic systems (Mackenzie, 2017; Pääkkönen et al., 2020), which nonetheless rarely perform their tasks perfectly when dealing with complex language data. As Grimmer and Stewart have noted (2013, p. 4), “[t]he complexity of language implies that all methods necessarily fail to provide an accurate account of the data-generating process used to produce texts.”

Differences emerge when observing the expectations for the technology in our empirical material: while the participants expressed dreams of magical and objective technological agency, the critical online discussions focused on fears of biased human agency embedded in the technology. Essentially, those who disagreed with the initial suggestion of countering hate speech perceived the implemented system as representative of undemocratic values. The critics, thus, acknowledged quite forthrightly the potential normativities and power structures embedded in algorithms (Ziewitz, 2016; Grosman and Reigeluth, 2019). The responses are indicative of an interesting dichotomy spanning the whole range of the project: while technology was used as a tool to deal with online emotions, simultaneously the responses tapped into the same emotions as a means to deal with the technological unknown, the mysterious algorithm conducting the monitoring or surveillance process. Those persons not a part of the project expressed frustration with the unknown and uncontrollable, while those that were a part of the project expressed frustration with the disappearing magic of technology, which failed to deliver on its mythical promises. Indeed, Mackenzie (2017) has pointed out that machine learning is not exactly about automation, but rather about a reconfiguration of the human-technology relationship. Implementing it generates new situations in which human knowledge becomes merged with probabilistic calculation, and the limitations of both are revealed.

Finally, our project deals with technologies that are increasingly being used in the industry, for example to monitor and moderate online discussions, and that will hence reshape our communication environments and our society in the future (see Brown, 2018). That is why we argue that a better understanding of the capabilities and limitations of these technologies is needed, and action research approaches and open science are some of the tools needed to generate public knowledge (see Kennedy et al., 2014). Our pilot project contributes to this need by opening the process of designing one such “black box” that builds on machine learning technologies and that in the industry would probably be marketed as a form of artificial intelligence. Furthermore, our case concerns a political wedge issue with great political and societal relevance, which effectively brings out affective sensemaking practices. While our project was a rather small-scale pilot, the inclusion of various organizational actors in the process allowed us to explore their sensemaking and expectations in the context of new technological solutions as well as to identify some relevant practical implications, which will be discussed next.

### Practical Implications

First and most importantly, a system that works to monitor hate speech or other forms of toxic language online should be a long-term, constant project with an *iterative approach* to its development. This requires initial, reliably annotated training data and a continuous flow of updated, human-annotated data for retraining the algorithm. The retraining loop in our system showed that the prediction scores became more accurate during the 1-month period. An iterative model could also solve some problems related to the known issue of context associated with machine learning models: developed models do not perform well if used in another, even slightly different, setting (Yu et al., 2008; Grimmer and Stewart, 2013). To some extent, the content flagging systems implemented and advocated by, for example, large technology companies (see European Commission, 2016) could be considered as a way to include the human factor in the loop. Such implementation would, for example, better account for the shifting nuances in the forms of soft hate speech and the periphrases and euphemisms being used. Another promising approach would be the use of transfer learning, as implemented for hate speech detection by Waseem et al. (2018).

Second, what makes the flagging systems risky is the fact that our findings highlight the importance of trained annotators with a good knowledge of both the phenomenon being classified as well as cultural connotations in relation to it; it is essential to be aware of local slur words and other expressions as well as any juridical definitions that the system may be based on. Similar notions have also been highlighted by the Rabat convention (OHCHR, 2013) and in the European Commission's 2018 recommendation: illegal online content should be identified in cooperation with trusted flaggers who are able to conduct a holistic evaluation of the content. One of the NGO interviewees stated that after participating in the project, it became evident that these kinds of projects should draw from multiple expertise areas: hate speech is a difficult concept for even for experts to define, so it would not be feasible to make individuals whose expertise is not in that area responsible for figuring out classification systems for this type of material. This means, for example, that crowdsourced annotations using MTurk or the like should be considered with great caution.

Third, despite the challenges related to the task we recommend that future hate speech recognition models not focus only on the content of the message, but that they also consider the contextual factors related to hate speech emphasized by various studies, recommendations, and definitions (e.g., OHCHR, 2013; Article 19, 2015; Gagliardone et al., 2015). These aspects include the broader discussion context of the message, the status and position of the poster of the message, and an evaluation of the publicity attracted by the message (see Rabat Action Plan, OHCHR, 2013, section 29). Existing technologies make it possible to, for example, identify the theme in the full online conversation, and APIs give numerical data on the message's reach. Such features could be implemented in detection models to give more contextual cues. However, including more sophisticated, ecologically valid features in models does not necessarily produce more usable models (Grimmer and Stewart, 2013). Therefore, such experimentation should be conducted with careful validation practices and human-computing approaches should also be considered. Finally, as the Rabat Action Plan (OHCHR, 2013, sections 35–40) also points out, legislation and technological procedures are only one form of countering hate speech and toxic online cultures. The Action Plan and the Article 19 Toolkit 2015 both stress the responsibility of political and religious leaders as well as the media, political parties, and other civil society actors to be ethically aware and socially responsible with respect to public speech.

Finally, a problematic aspect of any machine learning model is that the actual functionality of the model is difficult to explain to laypeople, who often nevertheless are subjected to the power of the model. As the online discussions that we observed in relation to the project show, people can be quite dubious of the design and implementation of algorithmic systems dealing with public discussion and easily regard them as a threat. One of the main questions thus is, how do we make such models more morally accountable? Transparency of the methods and training data—if not the full code and data or at least the principles of collecting and annotation—are essential. In this vein, transparency with respect to the people being monitored is also crucial, as are the legal basis and ethical considerations regarding the data collection in the first place—also required by the GDPR regulation and recommended by the European Commission (2018, sections 16–17). It has been noted that knowledge of the system principles can allow the instigators to bypass the system or move to other, less-regulated environments (e.g., Citron, 2014; Brown, 2018). However, we highlight, in accordance with the above-mentioned idea that reducing hate speech is also a social and ethical question, that more transparent models could be useful for educating people: working together with the users to build less toxic online cultures. However, increased openness also brings with it new kinds of responsibilities for the researchers and practitioners who are building systems that can be used for surveillance or profiling purposes. Therefore, it is essential to consider who will use the training data and algorithm after us and for what purposes.

## Data Availability Statement

The code generated in this project is available in the GitHub repository Automatic hate speech detection at https://github.com/futurice/spice-hate_speech_detection/.

## Ethics Statement

Ethical review and approval was not required for the study on human participants in accordance with the local legislation and institutional requirements. All interviewees gave written informed consent in accordance with the ethical principles of research with human participants given by the Finnish National Board on Research Integrity TENK.

## Author Contributions

S-ML led the manuscript writing process, designed the structure, wrote most parts of the text, member of the action research project team, took part in designing the machine learning part, preparing the classification scheme for the training data, and took part in the training data annotation. JH was responsible for planning and conducting the interviews as well as writing the analysis section and planning the manuscript structure together with S-ML. TK was responsible for designing and implementing the machine learning part, running the detection scripts during the project, writing the machine learning section in the manuscript, and part of the original project team. MN took part in designing the machine learning system, was responsible for streaming data collection during the project, took part in the training data annotation, wrote and commented machine learning related parts in the manuscript, and member of the original project team. RP wrote and assisted to write the sections concerning hate speech definitions and research, and helped to generate the definition and classification scheme for the original project.

### Conflict of Interest

TK is employed by the company Futurice Ltd, and the work for this study was conducted as a part of their social impact program Spice Program. The remaining authors declare that the research was conducted in the absence of any commercial or financial relationships that could be construed as a potential conflict of interest.

## Footnotes

^1^Criminal Code 19.12.1889/39. *Rikoslaki (Finnish Criminal Code)*. Available online at: https://www.finlex.fi/fi/laki/ajantasa/1889/18890039001

^2^Ethical Journalism Network (EJN) (n.d.). *Hate-Speech: A Five-Point Test for Journalists*. Available online at: https://ethicaljournalismnetwork.org/resources/publications/hate-speech (accessed September 13, 2019).
